# Updated Evidence of the Association Between Elevated Serum Uric Acid Level and Psoriasis

**DOI:** 10.3389/fmed.2021.645550

**Published:** 2021-06-29

**Authors:** Ying Zhang, Liu Liu, Xiaoying Sun, Hongjin Li, Yifei Wang, Min Zhou, Liang Hua, Bin Li, Xin Li

**Affiliations:** ^1^Department of Dermatology, Shanghai Skin Disease Hospital, Tongji University, Shanghai, China; ^2^Department of Dermatology, Yueyang Hospital of Integrated Traditional Chinese and Western Medicine, Shanghai University of Traditional Chinese Medicine, Shanghai, China; ^3^Institute of Dermatology, Shanghai Academy of Traditional Chinese Medicine, Shanghai, China

**Keywords:** psoriasis, hyperuricemia, meta-analysis, metabolic syndrome, obesity, gout

## Abstract

**Background:** Our earlier meta-analysis showed that the correlation between psoriasis and hyperuricemia might be region-dependent and that hyperuricemia was more common in patients with psoriasis in Western Europe. However, no further analysis could be conducted owing to the scarcity of data.

**Objective:** Our study aimed to further explore the association between psoriasis and hyperuricemia.

**Methods:** Six databases (PubMed, Embase, the Cochrane Central Register of Controlled Trials, the China National Knowledge Infrastructure database, the Chinese Scientific Journals Full Text Database, and the Wanfang Data Knowledge Service Platform) were searched for studies published between January 1980 and February 2021.

**Results:** The search strategy yielded 291 relevant studies, of which 27 observational studies were included in this analysis. Serum uric acid (SUA) levels (mean difference [MD] 0.99, 95% confidence interval [CI] 0.48–1.49, *P* = 0.0001) and hyperuricemia frequency (odds ratio [OR] 5.39, 95% CI 1.88–15.40, *P* = 0.002) were higher in the psoriasis group than in the control group, and the subgroup differences were significant. In addition, SUA levels were significantly higher in patients with moderate to severe psoriasis from European and American countries (MD 0.89, 95% CI 0.18–1.60, *P* = 0.01) and Southeast Asia (MD 1.79, 95% CI 0.55–3.02, *P* = 0.004), while no significant differences were found between the Middle East subgroup (MD 0.63, 95% CI −0.33 to 1.59, *P* = 0.20). Similar results were obtained from the meta-analysis of SUA levels in patients with metabolic syndrome, obesity, or a special type of psoriasis (such as arthritic or erythrodermic psoriasis).

**Conclusions:** Our meta-analysis study provides extended data regarding the correlation between psoriasis and hyperuricemia and the differences in SUA levels between psoriasis patients and controls in Southeast Asia, the Middle East, and European and American countries. Patients with moderate to severe psoriasis in European and American countries and Southeast Asia or those with metabolic syndrome and obesity were more likely to have higher uric acid levels.

**Systematic Review Registration:** PROSPERO, identifier: CRD42014015091.

## Introduction

Psoriasis is a chronic autoinflammatory disease that affects 2–4% of the population in Western countries (1, 2). It is characterized by keratinocyte hyperproliferation and increased epidermal cell turnover (3). Multiple mechanisms are involved in the pathogenesis of psoriasis, such as the presence of activated dendritic cells and T-lymphocytes, epidermal hyperproliferation, reduced keratinocyte differentiation, and oxidative stress (4–7).

Serum uric acid (SUA) mediates inflammatory pathways via the secretion of proinflammatory chemokines (8). Conversely, it has also been postulated that SUA acts as a potential antioxidant in patients with psoriasis (9, 10). Kwon et al. suggested that increased keratinocyte cell production induces an increase in purine metabolism, which elevates SUA levels in patients with psoriasis (11). According to surveys of SUA in adults in the United States, the normal range of SUA should be between 3.0 and 7.0 mg/dL (12). In 2016, we reported a systematic evaluation of the relationship between psoriasis and SUA level (13). Psoriasis is associated with various disorders such as cardiovascular diseases (14), metabolic syndrome (15, 16), obesity (17, 18), and chronic obstructive pulmonary disease (COPD) (19). Shared inflammatory pathways and common risk factors, such as obesity, glycometabolism disorders, and lipid metabolism disorders, may explain the pathogenesis of comorbidities in patients with psoriasis (2, 20). In a multivariate analysis, Gisondi et al. (18) identified psoriasis as the most important risk factor for hyperuricemia compared with other known risk factors such as obesity or metabolic syndrome. A meta-analysis by Li et al. (19) demonstrated that patients with psoriasis are at greater risk of developing COPD, and this association was stronger in patients with severe psoriasis. Elevated SUA levels have also been associated with outcomes related to metabolic syndrome, such as obesity, cardiovascular diseases, and hypertension. In another study, Li et al. (21) and Chen et al. (22) examined 136 unique associations between SUA level or SUA-lowering treatment and health outcomes and suggested five diseases highly associated with high SUA levels, namely heart failure, hypertension, impaired fasting glucose or diabetes, chronic kidney disease, and coronary heart disease. Considering the potential importance of uric acid, it is necessary to revise the systematic evaluation of the correlation between psoriasis and SUA levels to provide an updated data reference for relevant medical services and clinical interventions.

## Materials and Methods

### Data Sources and Searches

To identify relevant psoriasis studies in which SUA levels were measured as an outcome, three reviewers (YZ, LL, and XL) systematically searched six databases (PubMed, Embase, the Cochrane Central Register of Controlled Trials, the China National Knowledge Infrastructure database, the Chinese Scientific Journals Full Text Database, and the Wanfang Data Knowledge Service Platform) using the search terms psoriasis, hyperuricemia, and uric acid. Articles published between January 1980 ang February 2021 in English or Chinese were included in this study.

### Study Selection

**Figure 1:**
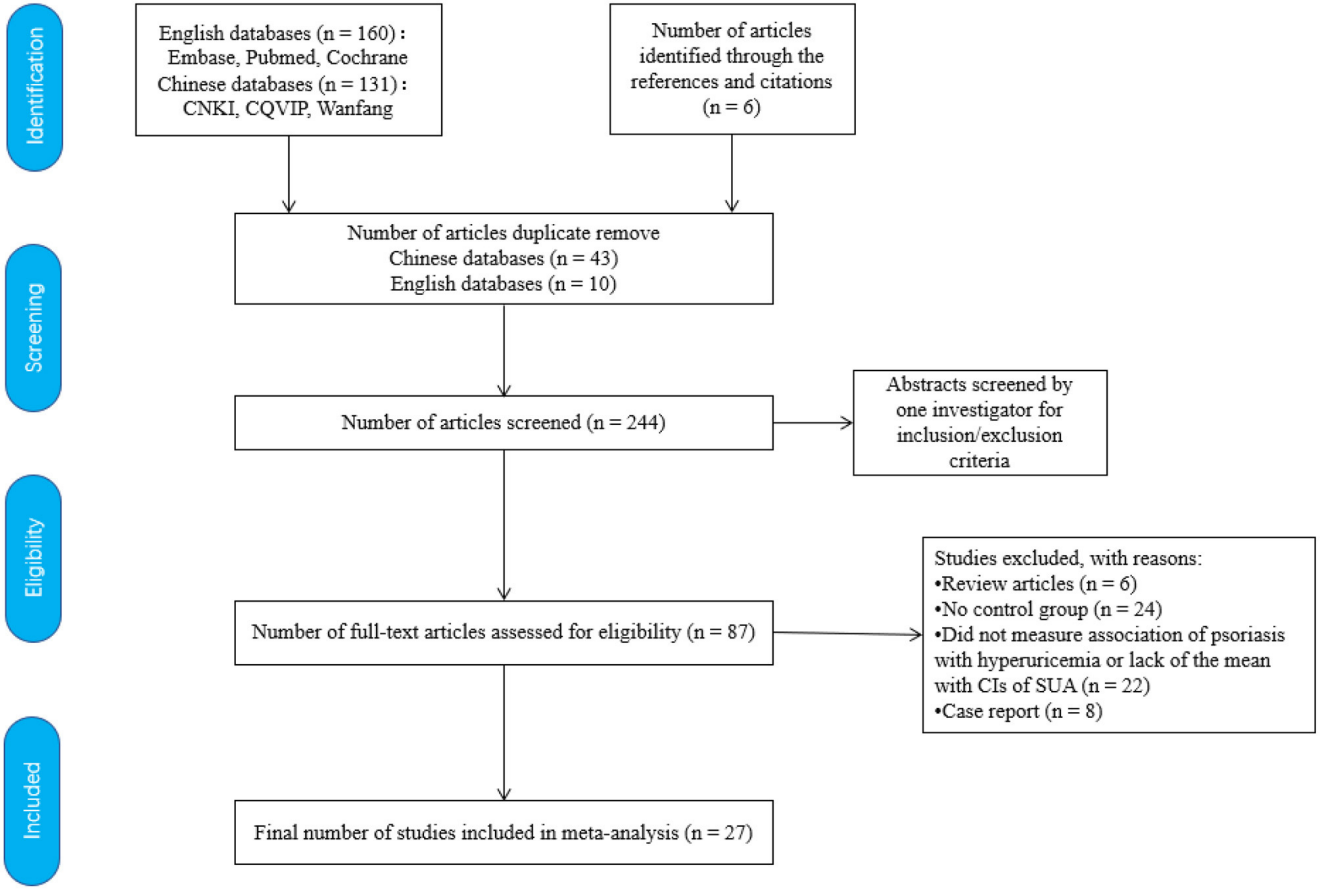
Flowchart of literature search and study selection process. CNKI, the China National Knowledge Infrastructure database; CQVIP, the Chinese Scientific Journals Full Text Database; Wanfang, the Wanfang Data Knowledge Service Platform; CIs, confidence intervals; SUA, serum uric acid.

The included publications were selected based on the following inclusion criteria: (1) human-only studies; (2) control group available; (3) original data available; and (4) estimation of the confidence intervals (CIs) by odds ratio (OR), risk ratio, hazard ratio (including enough data to calculate them), and hyperuricemia regarded as a specific outcome event or the mean CIs of SUA available. Initially, 291 articles were included in this study (Figure 1). A manual review of the references lists of the selected publications revealed six additional articles. We carefully reviewed these studies. Finally, 27 studies were included in this systematic review. A flowchart depicting the screening process is presented in Figure 1.

### Data Extraction and Quality Assessment

Three reviewers independently extracted the data using a predefined data extraction form for each included study. The extracted data included the first author, study characteristics, participant characteristics, and outcome characteristics. The Newcastle-Ottawa Scale (23) was used to assess study quality. Using this scale, the case-control studies were divided into selection, comparability, and exposure categories, while cohort studies were divided into selection, comparability, and outcome categories. The selection category contained four quality items: one for comparability and three for exposure.

### Data Synthesis and Analysis

The primary outcomes of this study were the differences between healthy controls and patients with psoriasis with respect to mean SUA levels and the relationship between psoriasis and the incidence of hyperuricemia. Cochrane's *X*^2^ and *I*^2^-tests were used to assess the degree of inter-study heterogeneity. Considerable heterogeneity was identified at *P* < 0.10 or *I*^2^-values above 50%. A random-effects model was used to compute the global OR and mean difference (MD). Fixed-effect models were used to examine studies with *P* > 0.05 or *I*^2^ < 50% or when the intra-study heterogeneity was not substantial. To analyze the possible reasons for the heterogeneity, we used prespecified variables and a random-effects meta-analysis for the subgroup analysis and meta-regression. The subgroups were analyzed based on the severity scales (such as Psoriasis Area and Severity Index [PASI]), sex, psoriasis type, and presence of metabolic disease. Prespecified sources of heterogeneity in the meta-regression included the source population, location, study design, study quality, psoriasis severity, presence of psoriatic arthritis, confirmed outcome, and outcome analysis. The methods and findings of the present review followed the guidelines and checklist of the Meta-analysis of Observational Studies in Epidemiology group (24). Review Manager 5.2 was used for the meta-analysis (http://ims.cochrane.org/revman). The meta-regression was performed using Stata version 15.0 (Stata Corp, College Station, TX, USA).

## Results

### General Characteristics and Methodology Assessment

Among the 297 identified studies, 27 were selected for inclusion in the systematic review and meta-analysis (Table 1). The meta-analysis consisted of two parts based on the type of response variable. The first part included the 27 studies (10, 11, 14–18, 25–44) that used continuous SUA as the response variable. These studies consisted of 31,314 participants (2,951 with psoriasis and 28,363 controls). The second part included seven studies (15, 17, 18, 29, 33, 36, 41) that had dichotomous hyperuricemia as the response variable. These studies included a total of 2,082 participants (958 with psoriasis and 1,124 controls) who met the inclusion criteria for the dichotomous variables of the systematic review. Of these 27 studies, 19 were cohort and eight were cross-sectional. Twenty-six studies provided means with CIs of SUA levels in psoriasis and control groups. Hyperuricemia was considered a specific outcome event, and ORs were provided in seven studies. Twenty studies suggested a statistically significant difference in SUA levels between psoriasis and control groups. Eleven studies evaluated outpatients. The studies were conducted in Southeast Asia, European and American regions, and the Middle East; among them, 17 were conducted in Asian countries.

**Table 1:** Observational studies included in the meta-analysis.

**Author (pub. year)**	**Study setting**	**Study period MM/ YY-MM/ YY**	**Study design**	**Outcome ascertainment**	**Measure of association (95%); mg/ dL**	**Controls: total number and number with hyperuricemia (%)**	**Cases: total number and number with hyperuricemia (%)**	**Age of controls, years, mean (SD)**	**Age of cases, years, mean (SD)**	**Cases receiving systemic therapy psoriasis**	**Use of acid-lowering drugs (%)**
Houshang et al. (10)	Iran; NR	NR	Prospective cohort	SUA	Control: 5.32 (0.95); Mild: 4.81 (0.63); Moderate: 4.67 (1.02); Severe: 4.44 (0.77)	Total: 100	Total: 100; Mild, 29; Moderate, 60; Severe, 11	35.7 (13)	Mild, 34 (9); Moderate, 37.6 (12); Severe, 35.5 (9)	All patients treated with topical agents only	NR
Gisondi et al. (18)	Italy; outpatient	01/ 12–06/ 12	Prospective cohort	Hyperuricemia: >6.0 mg/ dL females, >7.0 mg/ dL males	Control: 4.87 (1.4); Psoriasis: 5.61(1.6)	Total: 119; hyperuricemia: 8 (7)	Total: 119 hyperuricemia: 22 (19)	54.1 (12)	54.3 (8)	NR	NR
Ataseven et al. (25)	Turkey; NR	NR	Prospective cohort	SUA	Control: 4.20 (0.9); Psoriasis: 4.48 (1.2)	Total: 33	Total: 56	36.18 (14.19)	39.75 (18.29)	NR	NR
Alpsoy et al. (26)	Turkey; outpatient	08/ 10–10/ 12	Prospective cohort	SUA	Control: 4.2 (1.35); Psoriasis: 5.21 (1.44)	Total: 50	Total: 60	44.6 (11.3)	47.7 (13.4)	Methotrexate: 38; acitretin: 22	NR
Ibrahim et al. (14)	Egypt; outpatient	NR	Prospective cohort	SUA	Control: 4.44 (0.80); PsA: 5.75 (1.77)	Total: 60	PsA: 60; hyperuricemia: 14 (23.33)	48.97 (6.14)	48.90 (9.10)	Methotrexate mono- or combination therapy: 46; prednisone: 14	NR
Rajappa et al. (27)	India; NR	NR	Prospective cohort	SUA	Control: 4.81 (1.46); Psoriasis: 5.1 (1.59)	Total: 60	Total: 60.	43.75 (11.14)	41.97 (13.40)	Patients with PASI < 10 were receiving crude coal tar and liquid paraffin; those with PASI > 10 or PsA were receiving methotrexate	NR
Kwon et al. (11)	Korea; outpatient	Patients: 08/ 08–02/ 10; Control: 6,461 subjects 03/ 02–05/ 02; 20,000 6/ 93–03/ 94	Retrospective cross-sectional	Hyperuricemia: >6.0 mg/ dL females, >6.8 mg/ dL males	Control: 5.15 (1.40); Psoriasis: 5.1 (1.5)	Total: 26,461	Total: 198 Hyperuricemia: 38 (19.19)	NR	41.38 (14.39)	NR	NR
Isha et al. (28)	India; NR	NR	Prospective cohort	SUA	Control: 4.1 (0.19); skin disorder: 4.9 (0.19); psoriasis: 7.0 (0.64)	Control total: 25; skin disorder total: 25	Total: 25	Match	35.8	12 weeks of treatment	NR
Cassano et al. (29)	Italy; NR	NR	Prospective cohort	Hyperuricemia: >6.5 mg/ dL females, >7.0 mg/ dL males	Control: 5.3; psoriasis: 4.5	Total: 233; hyperuricemia: 13 (6)	Total: 146; hyperuricemia: 14 (10)	Match	52.5	NR	NR
Severin et al. (30)	Germany; inpatient	NR	Prospective cohort	SUA	Control: 5.47 (1.83); Severe: 6.69 (1.93)	Total: 36	Severe patients total: 33	NR	NR	NR	NR
Scott et al. (31)	England; outpatient	NR	Prospective cohort	SUA	Control: 4.93 (1.11); contact dermatitis: 5.19 (0.76); psoriasis: 5.23 (1.43)	Control total: 41; contact dermatitis total: 41	Total: 41	Control: 38; contact dermatitis: 41	38	NR	NR
Zhou et al. (32)	China; inpatient	NR	Retrospective case-control	SUA	Control: 4.71 (1.46); psoriasis: 5.66 (1.52)	Total: 57	Total: 292	Match with patient	NR	NR	NR
Zhang et al. (33)	China; outpatient or inpatient	03/ 06–03/ 11	Prospective cohort	SUA	Control: 3.44 (1.16); psoriasis: 6.56 (1.22)	Total: 286	Total: 307	37.71 (17.12)	48.56 (12.81)	NR	NR
Feng et al. (34)	China; outpatient or inpatient	NR	Prospectivecohort	SUA	Control: 4.78 (1.15); psoriasis: 4.84 (1.36)	Total: 65	Total: 293	NR	NR	NR	NR
Deng et al. (35)	China; outpatient	08/ 16–03/ 18	Prospective cohort	SUA	Control group: 4.71 (1.47); psoriasis group: 5.66 (1.54)	Total:70	Total: 230	48.13 (2.62)	47.37 (2.14)	NR	NR
Xie et al. (36)	China; outpatient	03/ 14–03/ 15	Prospective cohort	SUA	Control group (males): 6.63 (1.22), (females): 6.22 (1.03); psoriasis group (males): 3.82 (1.37), psoriasis group (females): 3.01 (1.17)	Total: 120; males: 5; females: 2; hyperuricemia: 7 (5.8)	Total: 120; males: 32; females: 14; hyperuricemia: 46 (38.3)	50.27 (3.29)	50.02 (3.10)	NR	NR
Lu et al. (37)	China; outpatient or inpatient	04/ 15–12/ 16	Prospective cohort	SUA	Control group: 4.35 (1.23); psoriasis group (blood heat type): 4.78 (1.46), psoriasis group (toxic heat type): 5.39 (1.10), psoriasis group (blood stasis type): 5.34 (1.32)	Total: 61	Total: 67; blood heat type: 27; toxic heat type: 18; blood stasis type: 22	45.1	43.2	NR	NR
Xia et al. (38)	China; inpatient	06/ 16–05/ 17	Prospective cohort	SUA, PASI	Control group: 5.56 (1.23); psoriasis group (PASI < 7): 5.95 (0.90), psoriasis group (PASI ≥ 7): 8.34 (0.89)	Total: 40	Total: 38; PASI < 7: 16; PASI ≥ 7: 22	41.72 (12.61)	39.42 (15.61)	NR	NR
Ge et al. (15)	China; outpatient	04/ 15–12/ 15	Retrospective cross-sectional	SUA	Control group: 4.87 (1.46); psoriasis group: 5.31 (1.34)	Total: 204; hyperuricemia: 14 (6.9)	Total: 204; hyperuricemia: 23 (11.3)	NR	42.29 (14.74)	NR	NR
Xu et al. (39)	China; inpatient	01/ 2001–12/ 2014	Retrospective cross-sectional	SUA	Control group: 5.15 (1.24); psoriasis vulgaris group: 5.71 (1.37); erythrodermic psoriasis group: 6.29 (1.92)	Total: 55	Psoriasis vulgaris: 62; erythrodermic psoriasis: 55	54.45 (14.32)	Psoriasis vulgaris: 51.16 (16.00); erythrodermic psoriasis: 55.11 (14.78)	NR	NR
Nicolae et al. (40)	Romania; NR	NR	Prospective cohort	SUA, PASI	Control group: 5.1 (0.4); psoriasis group (PASI < 7): 3.7 (0.6), psoriasis group (7 < PASI < 12): 5.1 (1.1), psoriasis group (PASI > 12): 5.7 (1.9)	Total: 45	Total: 45; PASI < 7:36; PASI (7 < PASI < 12): 5; PASI > 12: 4	39.4 (8.3)	40.38 (11.3)	NR	NR
Collazo et al. (41)	Mexico outpatient	11/ 14–01/ 15	Prospective cohort	SUA	Control group: 5.32 (1.17); psoriasis group: 7.03 (1.47)	Total: 45; hyperuricemia: 8 (17.88)	Total: 45; hyperuricemia: 31 (68.88)	50.98 (17.30)	49.38 (17.36)	NR	NR
Khan et al. (42)	Nepal; NR	NR	Retrospective cross-sectional	SUA, PASI	Control group: 5.22 (1.60); psoriasis group (PASI < 10):6.7 (1.38), psoriasis group (10 < PASI < 20): 6.75 (1.34), psoriasis group (PASI > 20): 6.88 (1.08)	Total: 50	Total: 50; PASI < 10: 41; PASI (10 < PASI < 20): 5; PASI > 20: 4	40.62 (9.72)	43.26 (10.62)	NR	NR
Gui et al. (17)	China; NR	NR	Retrospective cross-sectional	SUA	Control group: 5.71 (1.35), psoriasis group (PASI < 10): 5.5 (1.1), psoriasis group (PASI > 10): 6.3 (1.6)	Total: 117; hyperuricemia: 19 (16.24)	Total: 117; PASI < 10:112; PASI > 10:5; hyperuricemia: 37 (31.62)	NR	NR	NR	Allopurinol
Yilmaz et al. (16)	Turkey; NR	NR	Retrospective cross-sectional	SUA	Control group: 4.59 (1.26); psoriasis group: 5.08 (1.33)	Total: 70	Total: 70	44.73 (13.31)	42.24 (15.19)	NR	NR
Doǧan et al. (43)	Turkey; outpatient	03/ 14–08/ 15	Retrospective cross-sectional	SUA	Control group: 4.99 (1.27); psoriasis group: 5.63 (1.57)	Total: 73	Total: 199	44.93 (14.34)	43.62 (14.27)	NR	NR
Moustafa et al. (44)	Egypt; outpatient	NR	Prospective cohort	SUA, PASI, blood picture	Control group: 4.2 (1.25); psoriasis group (PASI < 10): 5.45 (1.875), psoriasis group (10 < PASI < 20): 5.65 (1.75), psoriasis group (PASI > 20): 6 (1.4)	Total: 20	Total: 60; PASI < 10: 20; PASI (10 < PASI < 20): 20; PASI > 20: 20	28.5 (17.25)	33.2 (16.25)	NR	NR

*MM/YY, month/year; NR, not reported; PASI, Psoriasis Area and Severity Index; PsA, psoriatic arthritis; SD, standard deviation; SUA, serum uric acid*.

### Quality Score of Included Studies

The Newcastle-Ottawa Scale scores ranged from 4 to 9 (Table 2). Among the included studies, 19 were considered of medium quality (4–6 stars) and eight were of high quality (≥7 stars).

**Table 2:** Newcastle-Ottawa Scale (NOS) quality assessment table.

**Study**	**Selection**	**Comparability**	**Exposure/ Outcome**	**Overall star rating**
Houshang et al. (10)	++	++	++	6
Gisondi et al. (18)	+++	++	++	7
Ataseven et al. (25)	++	++	++	6
Alpsoy et al. (26)	+	++	++	5
Ibrahim et al. (14)	++	++	++	6
Rajappa et al. (27)	+++	++	++	7
Kwon et al. (11)	++++		++	6
Isha et al. (28)	++	++	++	6
Cassano et al. (29)	+	++	++	5
Severin et al. (30)	++		++	4
Scott et al. (31)	++	++	++	6
Zhou et al. (32)	++	++	++	6
Zhang et al. (33)	++		++	4
Feng et al. (34)	++		++	4
Deng et al. (35)	++		++	4
Xie et al. (36)	++		++	4
Lu et al. (37)	+++	++	++	7
Xia et al. (38)	+++	++	++	7
Ge et al. (15)	+++	++	++	7
Xu et al. (39)	+++	++	++	7
Nicolae et al. (40)	++		++	4
Hernandez-Collazo et al. (41)	++	++	++	6
Khan et al. (42)	++	++	++	6
Gui et al. (17)	+	++	++	5
Yilmaz et al. (16)	+++	++	++	7
Doǧan et al. (43)	++	++	++	6
Moustafa et al. (44)	+++	++	++	7

*A star system was used to perform a semiquantitative assessment of study quality. In the selection and exposure categories, the study judged a maximum of 4 stars for each numbered item. A maximum of 2 stars can be given for comparability. The NOS ranges from 0 to 9 stars. High-quality studies achieved ≥7 stars, medium-quality studies achieved 4–6 stars, and poor-quality studies achieved <4 stars*.

### Primary Outcomes (Continuous Variables)

**Figure 2:**
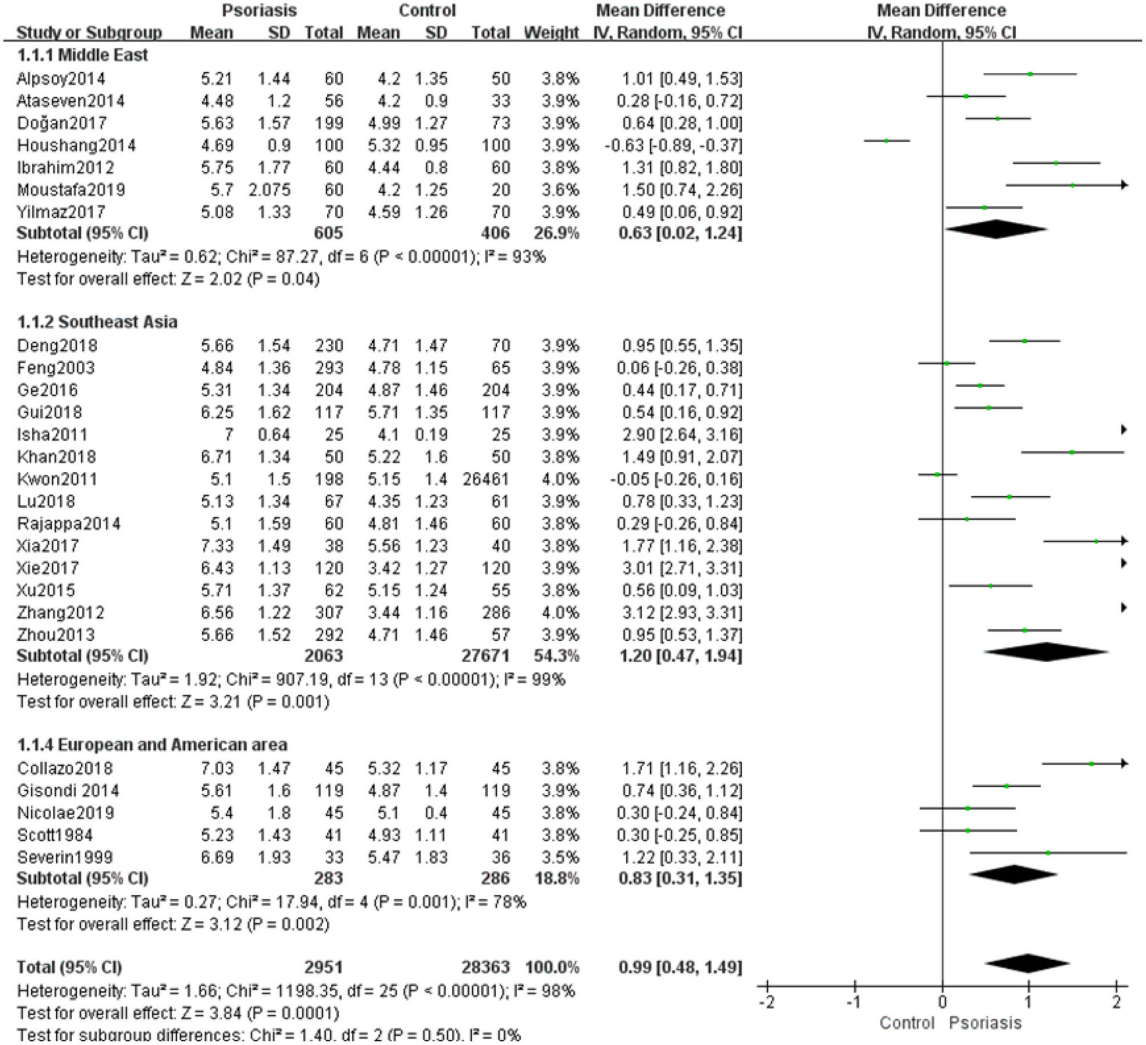
Meta-analysis of the SUA levels of patients with psoriasis vs. controls. The MD in SUA levels of patients with psoriasis compared with controls. The point estimate (center of each green square) and statistical size (proportional area of the square) are represented. The horizontal lines indicate the 95% confidence intervals. The subtotal and total MD (diamond) were calculated using a random-effects model. MD, mean difference; SUA, serum uric acid.

The meta-analysis of the SUA levels of patients with psoriasis showed significant inter-study heterogeneity (*I*^2^ = 98%, *P* < 0.00001). Specifically, the statistical heterogeneity among each subgroup was high (Middle East *I*^2^ = 93%, *P* < 0.00001; Southeast Asia *I*^2^ = 99%, *P* < 0.00001; and European and American regions *I*^2^ = 78%, *P* = 0.001). Using random-effects modeling, pooling of the results from these trials showed a significant difference in SUA levels between psoriasis patients and controls (MD 0.99, 95% CI 0.48–1.49, *P* = 0.0001). Although the heterogeneity was high, an optimal subgroup in Southeast Asia (MD 1.20, 95% CI 0.47–1.94, *P* = 0.001) was identified in a subgroup analysis. In addition, a significant difference was found in the European and American regions (MD 0.83, 95% CI 0.31–1.35, *P* = 0.002). Specifically, the difference between the Middle East groups almost reached statistical significance (MD 0.63, 95% CI −0.02 to 1.24, *P* = 0.04) (Figure 2). No statistically significant sources of heterogeneity were revealed in the meta-regression comparison of SUA levels between psoriasis patients and controls (Table 3).

**Table 3:** Potential prespecified sources of heterogeneity explored among studies reporting SUA levels of patients with psoriasis vs. controls.

**Prespecified source of heterogeneity**	**No. of studies**	**Random-effects meta-regression (95% CI)**	***P*-value**
Source population			0.876
Inpatient	6	0.879 (0.062–12.367)	
Outpatient	11	0.930 (0.295–2.932)	
Not clear	8	0.913 (0.250–3.330)	
Location			0.873
No	0	N/A	
Yes	24	0.813 (0.680–9.727)	
Not clear	2	0.667 (0.034–13.215)	
Study design			0.397
Cohort	18	0.945 (0.083-10.711)	
Cross-sectional	8	0.554 (0.045-6.779)	
Study quality			0.372
High (≥7 stars)	8	0.625 (0.049-8.005)	
Medium (4–6 stars)	18	0.894 (0.076-10.573)	
Poor (<4 stars)	0	N/A	
Severity of psoriasis			0.485
No distinction	16	0.926 (0.078–10.956)	
Mild vs. severe	10	0.634 (0.051–7.826)	
Psoriatic arthritis included			0.518
No	4	0.838 (0.052–13.568)	
Yes	6	0.960 (0.0653–14.113)	
Not clear	16	0.741 (0.057–9.648)	
Outcome ascertainment			0.786
Billing data	10	0.612 (0.050–7.46)	
Chart review	16	0.947 (0.081–11.05)	
Examination	0	N/A	
Analysis of outcome			0.249
Primary	18	1.008 (0.096–10.541)	
Secondary	8	0.473 (0.420–5.334)	

*CI, confidence interval; N/A, not applicable*.

### Secondary Outcomes

**Figure 3:**
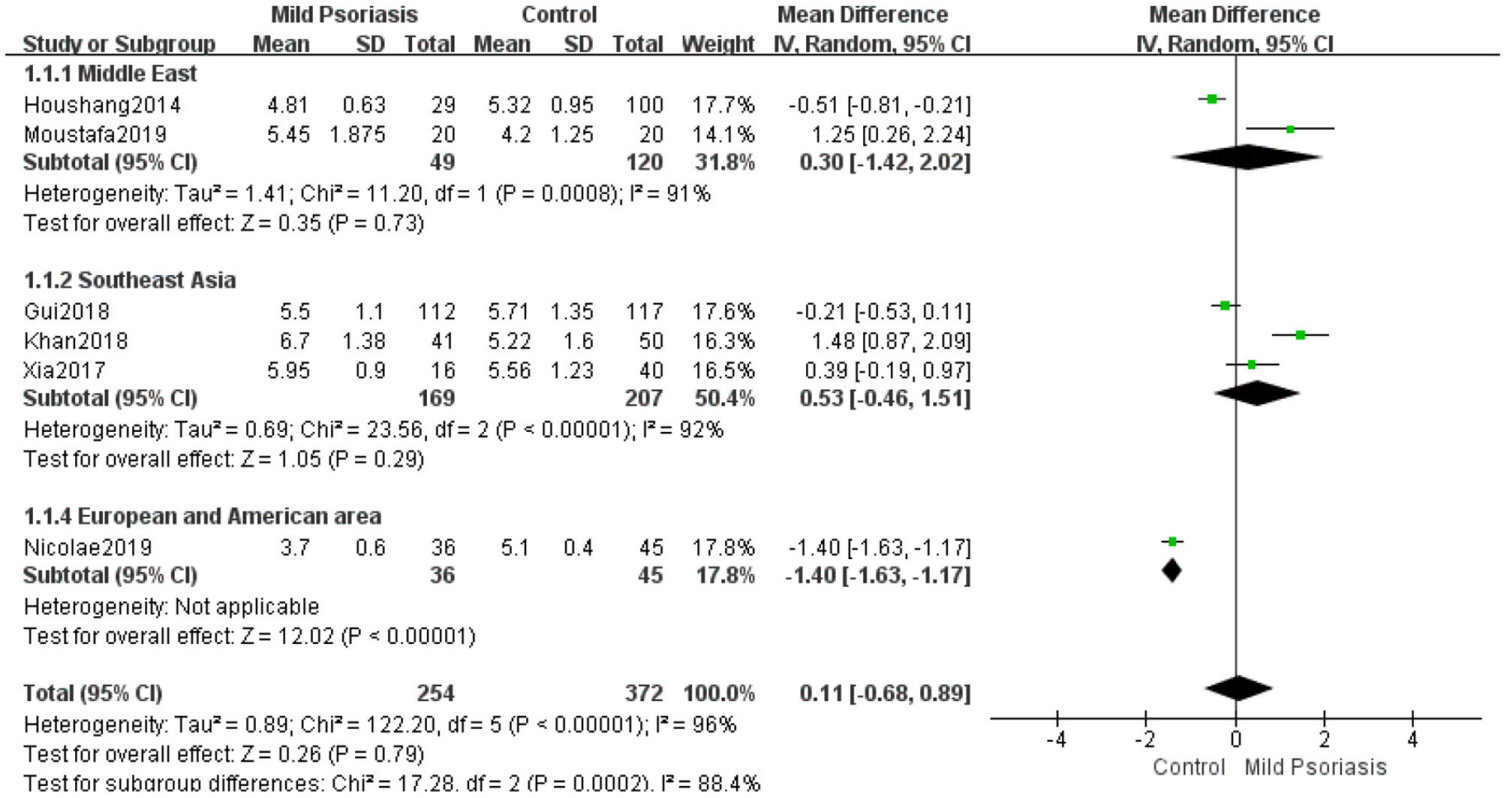
Meta-analysis of the SUA levels of patients with mild psoriasis vs. controls. The MD in SUA levels of patients with mild psoriasis vs. controls. The point estimate (center of each green square) and statistical size (proportional area of the square) are represented. The horizontal lines indicate the 95% confidence intervals. The subtotal and total MD (diamond) were calculated using a random-effects model. MD, mean difference; SUA, serum uric acid.

**Figure 4:**
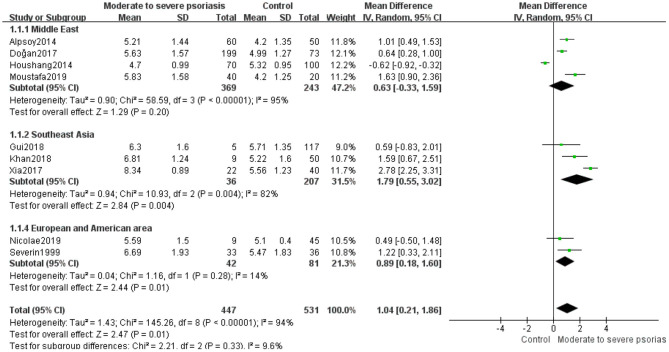
Meta-analysis of the SUA levels of patients with moderate to severe psoriasis vs. controls. The MD in SUA levels of patients with moderate to severe psoriasis vs. controls. The point estimate (center of each green square) and statistical size (proportional area of the square) are represented. The horizontal lines indicate the 95% confidence intervals. The subtotal and total MD (diamond) were calculated using a random-effects model. MD, mean difference; SUA, serum uric acid.

To determine SUA levels in patients with mild and moderate to severe psoriasis, we conducted a meta-analysis of significant inter-study heterogeneity in the mild psoriasis group (*I*^2^ = 96%, *P* < 0.00001) and the moderate to severe psoriasis groups (*I*^2^ = 94%, *P* < 0.00001). Using random-effects modeling, the pooled MD of five studies (10, 17, 38, 42, 44) in the mild psoriasis group was 0.11 (95% CI −0.68 to 0.89; *P* = 0.79). There was heterogeneity in the Middle East and Southeast Asian subgroups, but no significant differences were observed (MD 0.30, 95% CI −1.42 to 2.02, *P* = 0.73; MD 0.53, 95% CI −0.46 to 1.51, *P* = 0.29) (Figure 3). Using random-effects modeling, the pooled MD of nine studies (10, 17, 26, 30, 38, 40, 42–44) in the moderate to severe psoriasis group was determined as 1.04 (95% CI 0.21–1.86, *P* = 0.01). There was heterogeneity in the Middle East and Southeast Asian subgroups; in the European and American area subgroups, the heterogeneity was small (*I*^2^ = 14%, *P* = 0.28). Significant differences were found in the European and American area subgroups (MD 0.89, 95% CI 0.18–1.60, *P* = 0.01) and Southeast Asia subgroup (MD 1.79, 95% CI 0.55–3.02, *P* = 0.004), but not in the Middle East subgroup (MD 0.63, 95% CI −0.33 to 1.59, *P* = 0.20) (Figure 4). Taken together, these results suggest that in the moderate to severe psoriasis group, the significant difference in SUA levels between psoriasis patients and controls was likely to be clinically or regionally relevant and unlikely to be an artifact resulting from inter-study heterogeneity.

**Figure 5:**
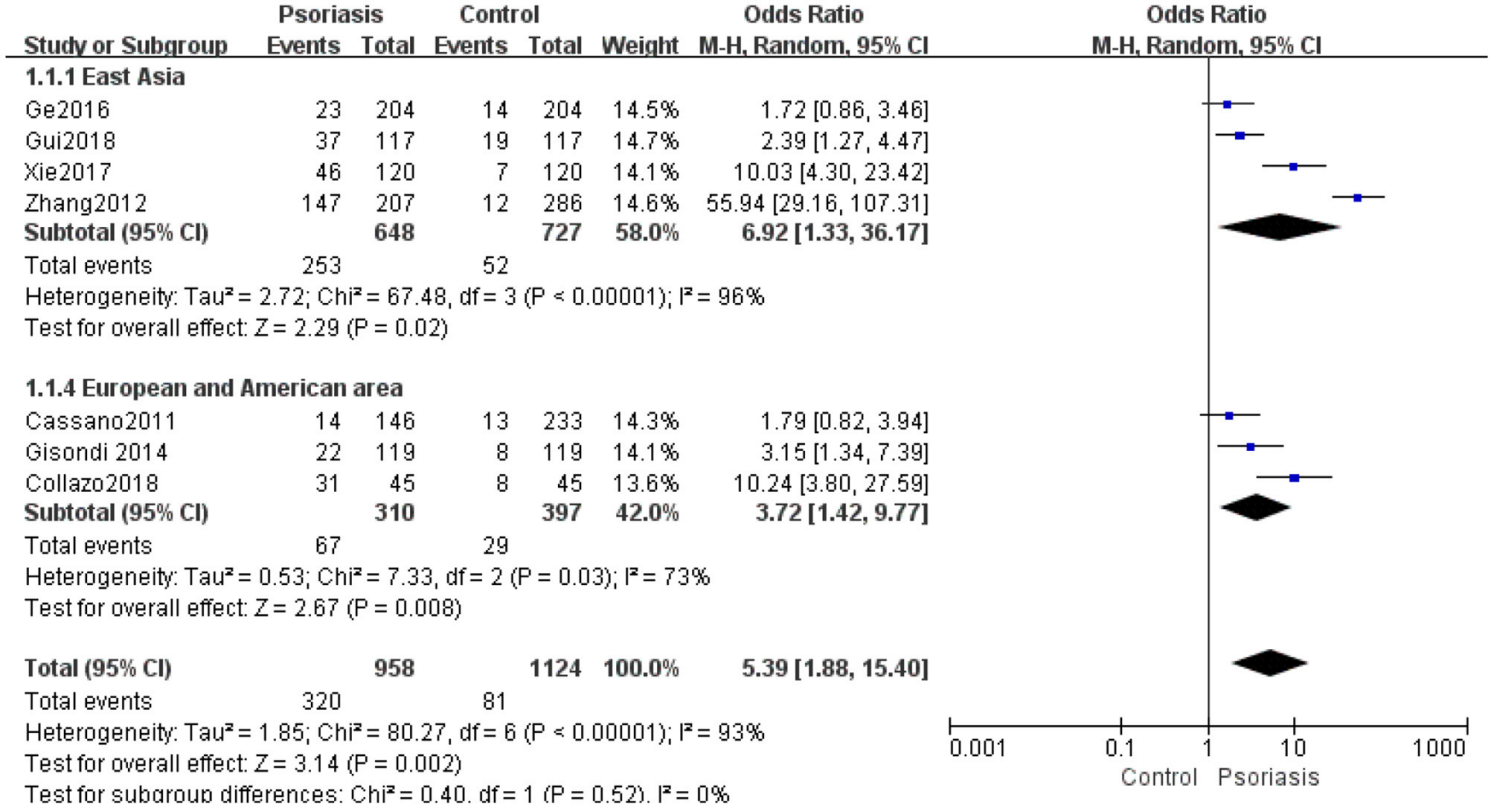
Meta-analysis of the prevalence of hyperuricemia in psoriasis patients vs. controls. OR for psoriasis in patients with hyperuricemia compared with total patients. The point estimate (center of each blue square) and statistical size (proportional area of the square) are represented. The horizontal lines indicate the 95% confidence intervals. The pooled OR (diamond) was calculated using a random-effects model. OR, odds ratio.

High inter-study heterogeneity existed in the meta-analysis of hyperuricemia occurrences among psoriasis patients and controls from the European and American regions and East Asia (*I*^2^ = 73%, *P* = 0.003). The pooled ORs from the random-effects analysis for seven studies (15, 17, 18, 29, 33, 36, 41) from European and American countries and East Asia, which revealed significant differences in hyperuricemia prevalence between psoriasis patients and controls, was 5.39 (95% CI 1.88–15.40, *P* = 0.002) (Figure 5).

**Figure 6:**
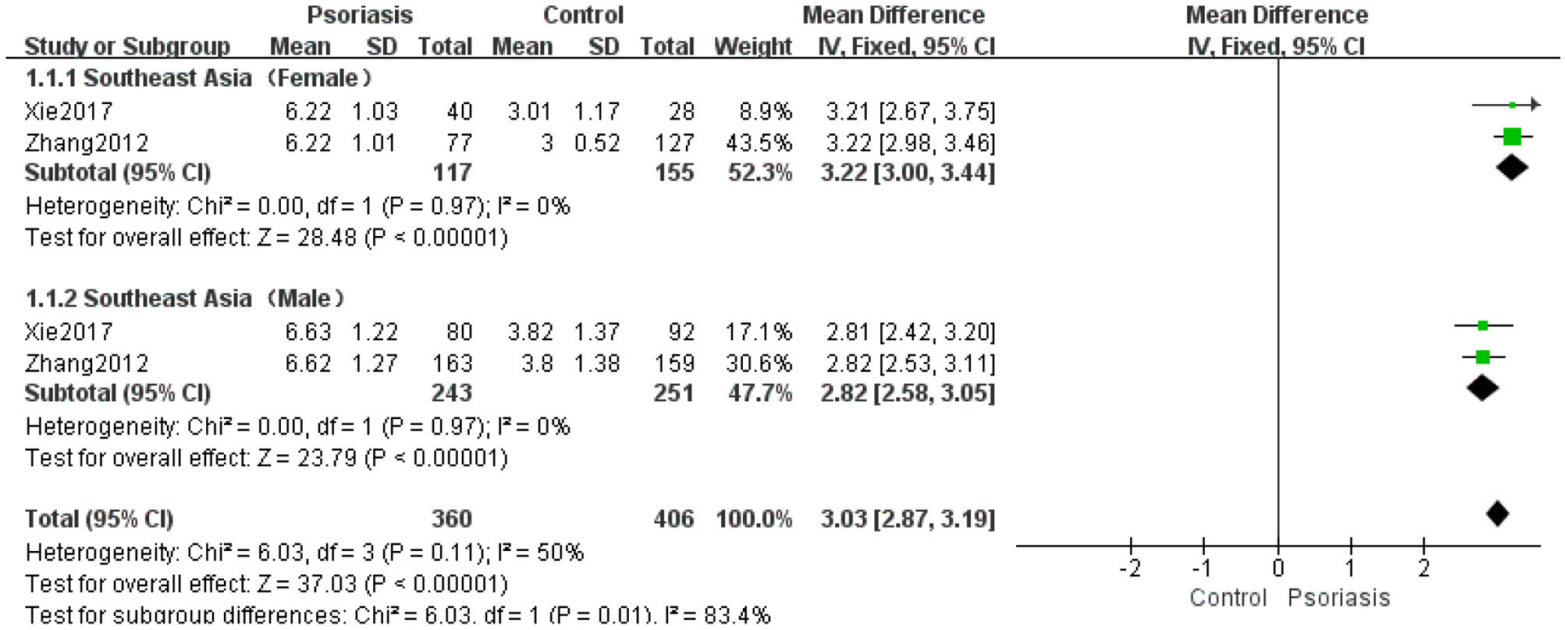
Meta-analysis of the SUA levels of psoriasis patients vs. controls by sex. The MD in SUA levels of psoriasis patients with different genders vs. controls. The point estimate (center of each green square) and statistical size (proportional area of the square) are represented. The horizontal lines indicate the 95% confidence intervals. The subtotal and total MD (diamond) were calculated using a fixed-effects model. MD, mean difference; SUA, serum uric acid.

Two studies (33, 36) compared SUA levels by sex between psoriasis patients and controls. The results of the meta-analysis using the fixed-effects model (*I*^2^ = 50%, *P* = 0.11) demonstrated little heterogeneity in the female and male subgroups, whereas a significant difference was observed between the two studies with a pooled MD of 3.03 (95% CI 2.87–3.19, *P* < 0.00001) (Figure 6).

**Figure 7:**
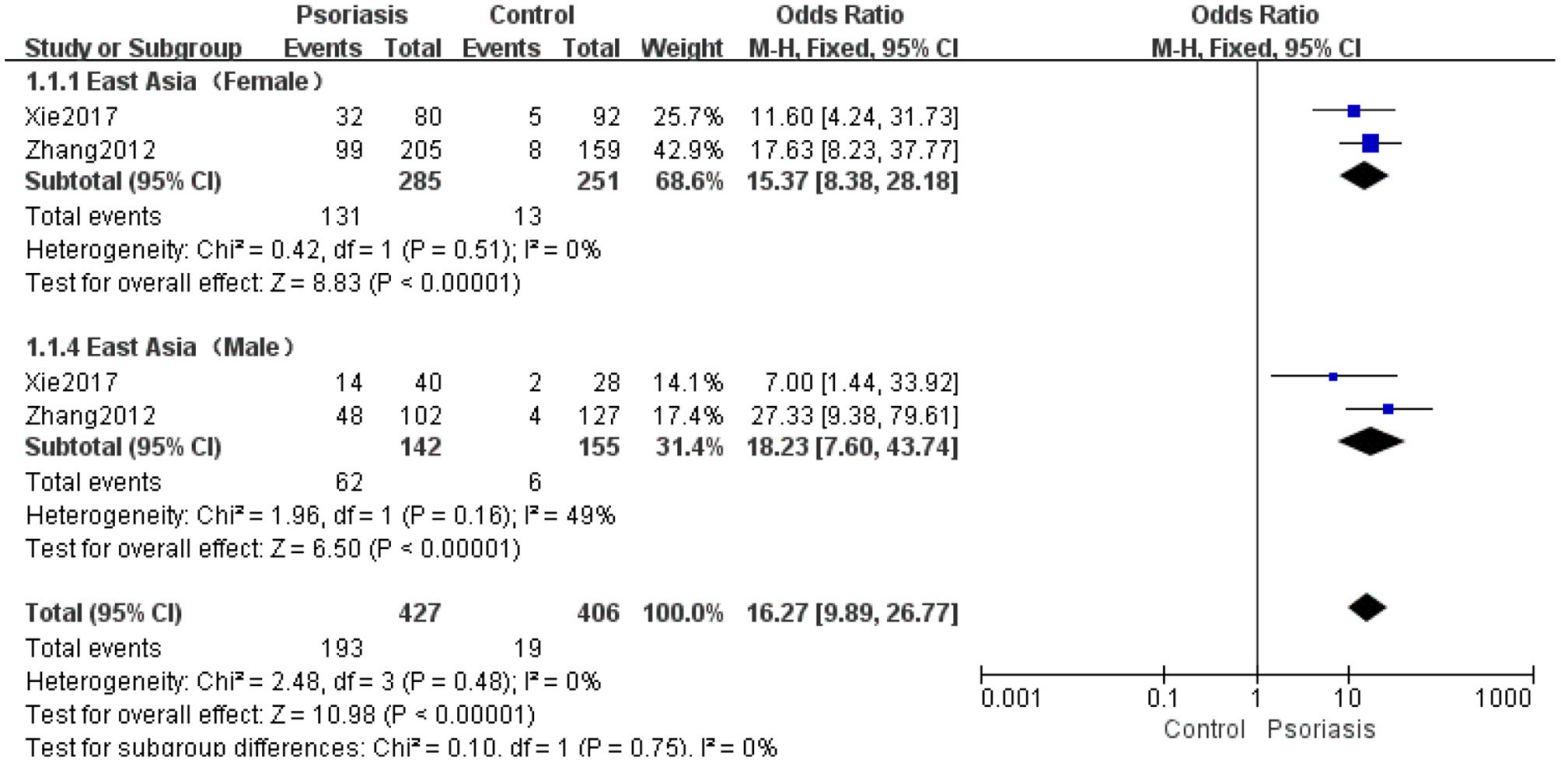
Meta-analysis of the prevalence of hyperuricemia in psoriasis patients vs. controls by sex. OR for psoriasis in subjects with hyperuricemia compared with total subjects. The point estimate (center of each blue square) and statistical size (proportional area of the square) are represented. The horizontal lines indicate the 95% confidence intervals. The pooled OR (diamond) was calculated using a fixed-effects model. OR, odds ratio.

Two studies (33, 36) analyzed the prevalence of hyperuricemia between the sexes in psoriasis patients and controls. The results of the meta-analysis using the fixed-effects model (*I*^2^ = 0%, *P* = 0.48) indicated that the hyperuricemia prevalence by sex differed significantly between psoriasis patients and controls (OR 16.27, 95% CI 9.89–26.77, *P* < 0.00001) (Figure 7).

**Figure 8:**
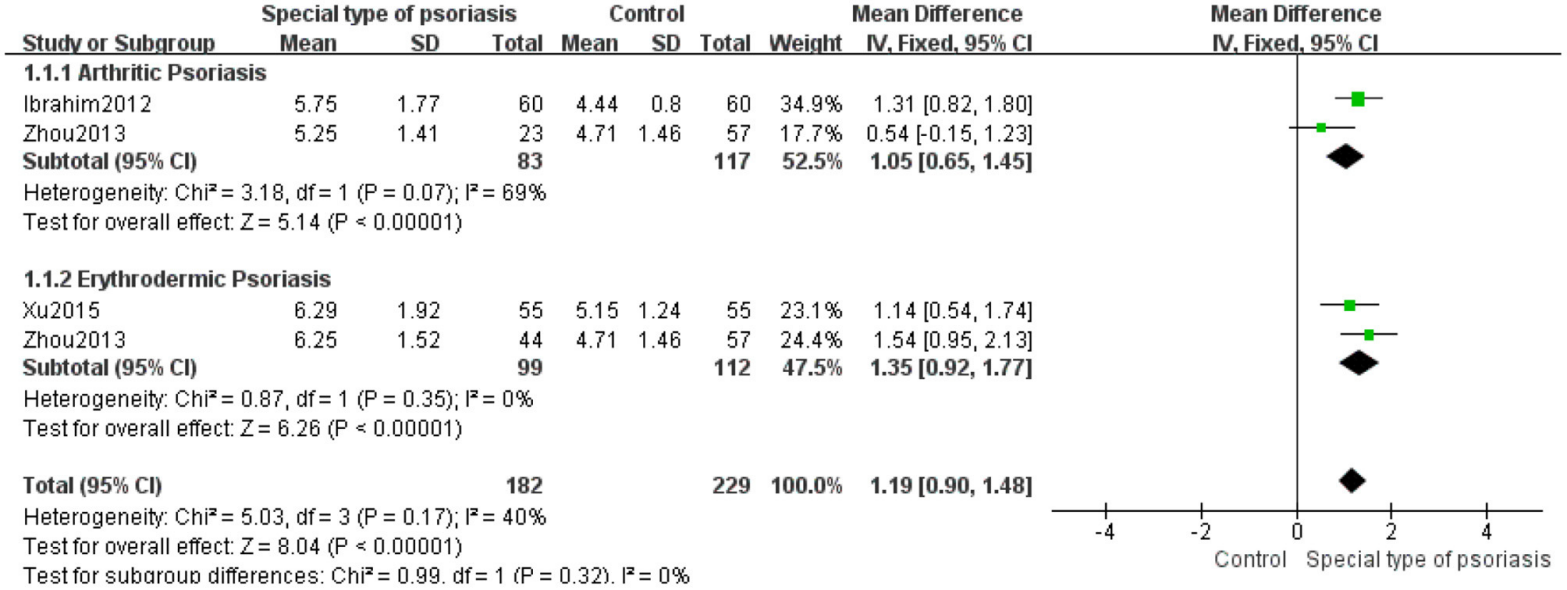
Meta-analysis of the SUA levels of patients with a special type of psoriasis vs. controls. The MD in SUA levels of patients with a special type of psoriasis compared to controls. The point estimate (center of each green square) and statistical size (proportional area of the square) are represented. The horizontal lines indicate the 95% confidence intervals. The subtotal and total MD (diamond) were calculated using a fixed-effects model. MD, mean difference; SUA, serum uric acid.

Analysis of SUA levels in each subgroup of patients with a special type of psoriasis like arthritic (14, 36) or erythrodermic (32, 39) showed significant differences between the subgroups (MD 1.19, 95% CI 0.90–1.48, *P* < 0.00001) using the fixed-effects model (*I*^2^ = 40%, *P* = 0.17). There was heterogeneity in arthritic psoriasis subgroup (*I*^2^ = 69%, *P* = 0.07), little heterogeneity in the subgroup of erythrodermic psoriasis (*I*^2^ = 0%, *P* = 0.35), and significant differences were found in both subgroups (MD 1.05, 95% CI 0.65–1.45, *P* < 0.0001; MD 1.35, 95% CI 0.92–1.77, *P* < 0.00001) (Figure 8).

**Figure 9:**
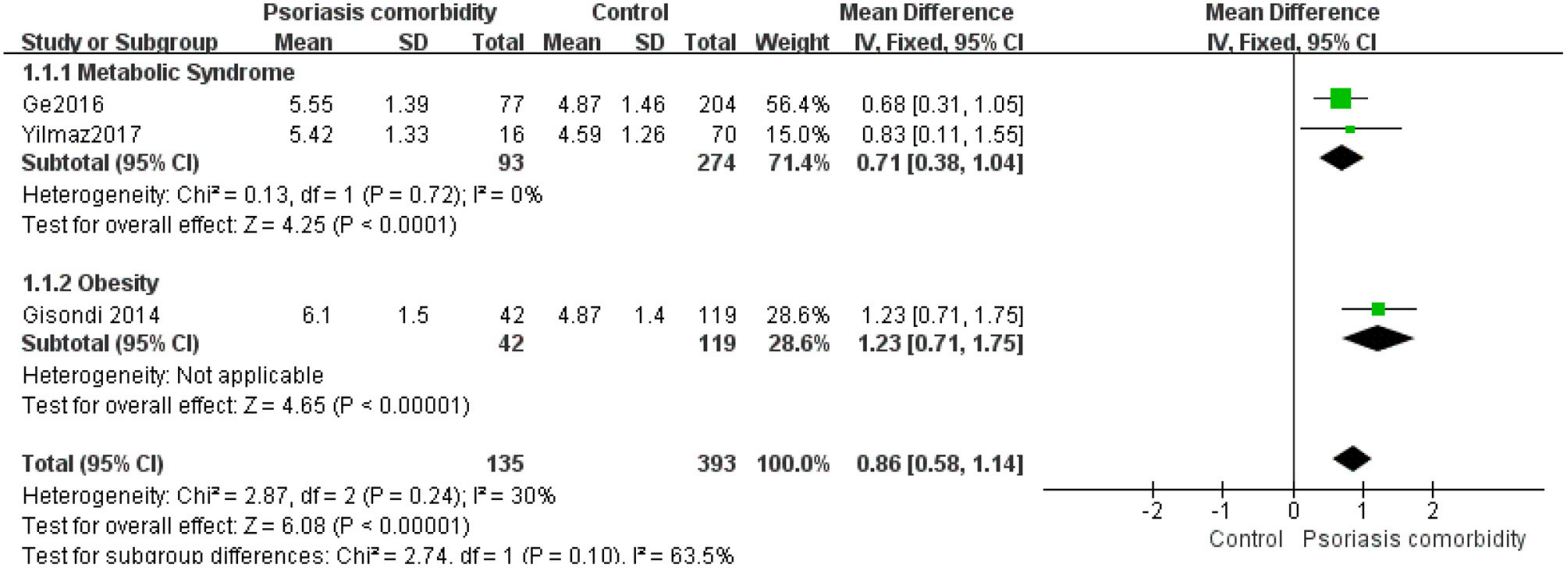
Meta-analysis of the SUA levels of psoriasis patients with comorbidities vs. controls. The MD in SUA levels of psoriasis patients with comorbidities compared to controls. The point estimate (center of each green square) and statistical size (proportional area of the square) are represented. The horizontal lines indicate the 95% confidence intervals. The subtotal and total MD (diamond) were calculated using a fixed-effects model. MD, mean difference; SUA, serum uric acid.

A meta-analysis of SUA levels among patients with psoriasis who had metabolic syndrome (15, 16) or obesity (18) and controls revealed low inter-study heterogeneity (*I*^2^ = 30%, *P* = 0.24). Using fixed-effects modeling, we observed significant differences in SUA levels between psoriasis patients with metabolic syndrome or obesity and controls in three studies (15, 16, 18), with a pooled MD of 0.86 (95% CI 0.58–1.14, *P* < 0.00001) (Figure 9).

## Discussion

Psoriasis is a skin disease that affects people worldwide. While its pathogenesis is unclear, it is believed to be related to several factors, such as heredity, immunity, and environmental factors (16). Clinical research has recognized a correlation between the increase in SUA level and psoriasis (14). In our previous meta-analysis, we assessed data from 14 clinical trials (10, 11, 14, 18, 25, 27–34, 43) including 29,416 patients with psoriasis and concluded that the correlation between psoriasis and hyperuricemia showed a regional pattern and was associated with genetic components and lifestyle differences (13). In this study, we expanded the data from the earlier study to confirm the correlation between psoriasis and hyperuricemia and reveal the potential relevance between morbidity related to some cardiovascular or metabolic diseases and SUA elevation in psoriasis patients.

This meta-analysis combined data from 27 studies that covered most of the available global epidemiological evidence for psoriasis. We analyzed SUA levels in psoriasis patients among different regions, sexes, PASI scale scores, and psoriasis types as well as the presence or absence of metabolic disease. SUA levels and hyperuricemia frequency were significantly higher in the psoriasis group than in the control group. Similar results were obtained when patients were stratified by region, sex, and metabolic disease. In addition, SUA levels in erythrodermic psoriasis and arthritic psoriasis were higher than those in the control group. The MD value of 0 was outside the 95% CI of SUA level in the three subgroups (European and American regions, Southeast Asia, and the Middle East). In seven studies (11, 15, 18, 39, 40, 43, 44), a significant correlation was observed between SUA levels and PASI scores, whereas five studies (27, 28, 34, 36, 42) presented contradictory results. In our study, the stratification of psoriasis patients by PASI score into the mild and moderate to severe psoriasis subgroups revealed that moderate to severe psoriasis patients from European and American regions and Southeast Asia were more likely to have hyperuricemia. No significant correlation was observed between patients with mild psoriasis from the European and American regions, Southeast Asia, or the Middle East and hyperuricemia.

Analysis of the seven studies (15, 17, 18, 29, 33, 36, 41) included in the second part of the meta-analysis showed that the risk of hyperuricemia was high in psoriasis patients. Two studies that performed a subgroup analysis based on sex (33, 36) indicated a significant difference in the prevalence of hyperuricemia between the psoriasis and control groups.

Additionally, the results of this study suggest that elevated SUA levels or hyperuricemia was positively associated with metabolic syndrome (15, 16) and obesity (18). Unfortunately, no cardiovascular disease was mentioned by any of the included studies. Psoriasis is reportedly an independent risk factor for cardiovascular diseases (29, 41). However, the relationship between SUA levels and cardiovascular events in psoriasis is unknown. Ibrahim et al. (14) suggested that chronic systemic inflammation and endothelial dysfunction appear to be the link between asymptomatic hyperuricemia and atherosclerosis. Cassano et al. (29) indicated a trend toward a correlation between SUA level and cardiovascular risk profile in psoriasis patients. Consequently, SUA may be a new marker of atherosclerosis and cardiovascular events in psoriasis patients. Furthermore, high SUA levels are associated with outcomes related to metabolic syndromes, such as obesity and cardiovascular diseases. Yilmaz et al. (16) studied SUA levels in 70 psoriasis patients and determined that SUA levels were higher in patients with psoriasis and metabolic syndrome than in those with psoriasis alone. Obesity is a critical comorbidity of psoriasis. Gisondi et al. (18) reported that SUA levels were significantly higher in obese patients than in non-obese patients with psoriasis. Zheng et al. (45) highlighted the fact that intense physical activity lowered the prevalence of psoriasis by reducing fat accumulation. Our meta-analysis showed that SUA levels were significantly higher in patients with moderate to severe psoriasis in European and American regions and Southeast Asia. Thus, it is worth exploring whether metabolic diseases and the clinical severity of patients with psoriasis are positively correlated in these regions. Therefore, the incidence of metabolic syndrome components should be given more attention when SUA levels increase in psoriasis patients, especially those with high PASI scores.

Notably, SUA levels of patients with arthritic psoriasis or erythrodermic psoriasis were significantly higher than those of controls. However, only one study (15) reported a positive correlation between SUA levels and erythrodermic psoriasis, similar to arthritic psoriasis (33). Elevated SUA level are commonly associated with psoriasis (14), and hyperuricemia predisposes patients to gout arthritis (46). However, the distinction between psoriatic arthritis associated with hyperuricemia and gouty arthropathy with psoriasis is complex. In our study, there were no prospective data on the association between psoriasis and gout. Felten et al. (47) suggested a new line of thinking regarding the convergence of gout and psoriatic arthritis that involved the role of urate crystals, which could prompt a potential new treatment approach (urate-lowering therapy) for refractory psoriatic arthritis.

We evaluated the effects of multiple variables on the meta-regression findings among the included studies by performing a meta-regression analysis to determine the factors underlying high heterogeneity but found no statistically significant difference in outcomes.

In conclusion, the current meta-analysis provided extended data regarding the correlation between psoriasis and hyperuricemia and indicated a difference in SUA levels between psoriasis patientsand controls in Southeast Asia, the Middle East, and European and American regions. Psoriasis patients from East Asia, Europe and America have higher SUA levels and rates of hyperuricemia. Meanwhile, patients with moderate to severe psoriasis in European and American regions and Southeast Asia or those with metabolic syndrome and obesity were more likely to have higher uric acid levels. Increasing attention is being paid to psoriasis comorbidities. The topic of psoriasis comorbidities was reviewed in detail in the 2018 Joint AAD-NPF guidelines (20). Future investigations are needed to establish a well-connected network between comorbidities in psoriasis and hyperuricemia or SUA levels and to prevent further comorbidities as well as disease progression. However, due to the inclusion of large numbers of cross-sectional design and cohort design studies in this meta-analysis, it was not possible to draw any conclusions about causality between psoriasis and elevated SUA levels or hyperuricemia. Nonetheless, all patients with psoriasis should undergo SUA screening by their healthcare providers according to national guidelines, with increased frequency considered for patients with obesity, high PASI scores, and asymptomatic hyperuricemia since elevated SUA levels may play a significant role in the progression of metabolic syndrome, cardiovascular disease, and gout in these patients.

## Data Availability Statement

The original contributions presented in the study are included in the article/Supplementary Material, further inquiries can be directed to the corresponding author/s.

## Author Contributions

YZ, LL, XS, HL, YW, MZ, and LH had full access to all study data and take responsibility for the analytical integrity and accuracy. YZ, LL, XL, and BL were responsible for the study concept and design. YZ and LL were responsible for the data acquisition. XS, HL, YW, and MZ managed the data extraction. YZ and XL performed the assessment of bias risk. YZ, LL, LH, and XL performed the data analysis and interpretation. YZ drafted the manuscript. LL, XL, and BL, while YZ, LL, XL, and BL provided a critical manuscript review for important intellectual content. YZ and LL performed the statistical analyses. XL and BL supervised the study. All authors contributed to the article and approved the submitted version.

## Conflict of Interest

The authors declare that the research was conducted in the absence of any commercial or financial relationships that could be construed as a potential conflict of interest.

## Abbreviations

AAD-NPF: American Association of Dermatologists and the National Psoriasis Foundation
CI: confidence interval
COPD: chronic obstructive pulmonary disease
MD: mean difference
OR: odds ratio
PASI: Psoriasis Area and Severity Index
SUA: serum uric acid.
